# Exploring Biophysical Linkages between Coastal Forestry Management Practices and Aquatic Bivalve Contaminant Exposure

**DOI:** 10.3390/toxics9030046

**Published:** 2021-03-02

**Authors:** Kaegan Scully-Engelmeyer, Elise F. Granek, Max Nielsen-Pincus, Andy Lanier, Steven S. Rumrill, Patrick Moran, Elena Nilsen, Michelle L. Hladik, Lori Pillsbury

**Affiliations:** 1 Department of Environmental Science and Management, Portland State University, Portland, OR 97201, USA; graneke@pdx.edu (E.F.G.); maxnp@pdx.edu (M.N.-P.); 2 Oregon Department of Land Conservation and Development, Salem, OR 97301, USA; andy.lanier@state.or.us; 3 Oregon Department of Fish and Wildlife, Newport, OR 97365, USA; Steven.S.Rumrill@state.or.us; 4 U.S. Geological Survey, Washington Water Science Center, Tacoma, WA 98402, USA; pwmoran@usgs.gov; 5 U.S. Geological Survey, Oregon Water Science Center, Portland, OR 97201, USA; enilsen@usgs.gov; 6 U.S. Geological Survey, California Water Science Center, Sacramento, CA 95819, USA; mhladik@usgs.gov; 7 Oregon Department of Environmental Quality, Hillsboro, OR 97124, USA; lori.pillsbury@state.or.us

**Keywords:** agrochemical, clam, ecotoxicity, fungicide, herbicide, multiple stressors, mussels, oyster, pesticide

## Abstract

Terrestrial land use activities present cross-ecosystem threats to riverine and marine species and processes. Specifically, pesticide runoff can disrupt hormonal, reproductive, and developmental processes in aquatic organisms, yet non-point source pollution is difficult to trace and quantify. In Oregon, U.S.A., state and federal forestry pesticide regulations, designed to meet regulatory water quality requirements, differ in buffer size and pesticide applications. We deployed passive water samplers and collected riverine and estuarine bivalves *Margaritifera falcata*, *Mya arenaria*, and *Crassostrea gigas* from Oregon Coast watersheds to examine forestry-specific pesticide contamination. We used non-metric multidimensional scaling and regression to relate concentrations and types of pesticide contamination across watersheds to ownership and management metrics. In bivalve samples collected from eight coastal watersheds, we measured twelve unique pesticides (two herbicides; three fungicides; and seven insecticides). Pesticides were detected in 38% of bivalve samples; and frequency and maximum concentrations varied by season, species, and watershed with indaziflam (herbicide) the only current-use forestry pesticide detected. Using passive water samplers, we measured four current-use herbicides corresponding with planned herbicide applications; hexazinone and atrazine were most frequently detected. Details about types and levels of exposure provide insight into effectiveness of current forest management practices in controlling transport of forest-use pesticides.

## 1. Introduction

Coastal zone management has evolved into a complex and multidisciplinary framework incorporating management priorities and considerations beyond the shoreline to include processes and conditions in adjacent terrestrial and riverine environments [1,2]. This approach relies on sufficient physical and socioecological knowledge of land–sea connections to understand cross-ecosystem threats to coastal and marine resources and guide management decisions that protect ecosystem functions [3,4]. Additional research and case-history investigations are needed to better understand how diverse land uses affect coastal species and ecosystems [5].

Oregon’s coastal zone, on the West Coast of the United States, encompasses the state’s coastal watersheds and extends approximately three miles seaward into nearshore marine waters [6]. Oregon coastal watersheds are largely forested and managed under several forestry management regimes [7], with the exception of Christmas tree farms, sporadic lowland agricultural lands, and rural towns and communities scattered throughout the region. The orientation of multiple small coastal watersheds along the linear Oregon coast, coupled with broad similarity in local climatic conditions, presents an opportunity to develop comparative case histories that explore effects of forestland management practices on coastal watersheds under contrasting management regimes.

Empirical investigations have found significant relationships between the scale of actively managed forestlands and cumulative effects on downstream water quality and quantity within watersheds [8,9]. Despite substantial research effort on cumulative effects of many forestry practices (such as road building, clearcutting, planting, and thinning), little is known about cumulative effects from multiple applications of chemical mixtures within watersheds and their transport away from the primary application site [10,11]. Most research on chemical applications and development of germane best management practices (BMPs) is focused on site-level effectiveness; this study aims to address lingering data gaps on the effects of chemical applications across multiple catchments on the fate and transport of compounds and mixtures.

### 1.1. Forest Management in Oregon’s Coastal Zone

Oregon’s forests are managed under two regulatory regimes: (1) federal lands, regulated under the Northwest Forest Plan (NWFP); and (2) private, industrial, state, and tribal lands, regulated under Oregon’s Forest Practices Act (OFPA), with Oregon’s State Forestry Management Plan building upon OFPA to offer additional protections and management objectives within state forests. Each plan prescribes a set of BMPs to guide activities such as timber harvest, pesticide use and application, road construction, and riparian vegetated buffer retention for each land ownership type.

NWFP implementation in 1994 instituted a dramatic shift in forestry management on federal lands throughout the Pacific Northwest region as the ecosystem-based management (EBM) approach extended management considerations beyond timber production [12]. Revised management objectives on federal lands resulted in significant portions of federal forestland being taken out of harvest rotation and allocated for other uses such as conserving biological diversity and endangered species [12,13]. Since NWFP implementation, state-regulated forests, including private and industrial forestlands, have comprised the majority of regeneration harvest, vegetation management, reforestation, and stand management (collectively known as intensive forest management; IFM) [14,15]. IFM activities on private, industrial, and tribal land are subject to the Forest Practices Administrative Rules under the OFPA. Hardwood timber, orchard trees, and Christmas tree production are designated as agriculture rather than timber operations under state law and therefore not subject to prescriptive regulations under the OFPA [16].

Though BMPs are designed to guide activities under both plans to meet federal regulatory requirements for the Endangered Species Act (ESA) listed species and Clean Water Act (CWA) water quality guidelines, differences in stream protections between federal and state standards vary substantially, including clear differences in vegetated buffer protections and chemical application guidelines in coastal watersheds [16,17]. For example, riparian management area (RMA) designations, which are generally determined by stream size, flow duration, fish presence, and/or domestic water usage, vary widely among land ownership types, with the largest protections on federal lands (30~152 m), followed by state (7.6–52 m) and private/industrial lands (0–30.5 m) [16]. Furthermore, at the time of the study, foresters operating under the OFPA in the Coast Range are generally not required to establish chemical-free buffers for aerial or ground spray adjacent to headwater streams classified as small non-fish-bearing, intermittent, or ephemeral, though new regulations now require an 18.3 m buffer if the stream is flowing at the time of application (OAR × 629–640–0400) [18,19]. In contrast, on federal lands in Oregon west of the Cascade Mountain Range, aerial application of herbicides for tree production is not permitted [20].

### 1.2. Chemical Applications in Forestry Practices

Contemporary IFM relies on numerous chemical products to re-establish and maintain tree plantations by managing competitive native and non-native vegetation and controlling pests that interfere with seedling or plantation success [21]. Spring and late summer/fall are the most common seasons for herbicide treatments in Oregon IFM, as application timing and effectiveness is prompted by phenological cues associated with conifer tolerances and target plant vulnerability [21].

Increased complexity and specificity of forest management areas coupled with increased diversity and targeting of chemical applications has led to over 900 chemical products comprising over 200 active ingredients currently registered for use in Oregon’s conifer forests [22]. Since the molecular formulations of these chemical compounds are targeted to control a specific type or suite of species, managers commonly use mixtures to maximize effectiveness of chemical application events [10].

The state of Oregon and federal agencies each have reporting systems to track pesticide applications on timberlands within their jurisdiction. Planned management actions on state, private, and tribal lands must be submitted to the Forestry Activity Electronic Reporting and Notification System (FERNS), which provides a record of approved activities and their locations. Management activities on federal lands are recorded by the U.S. Forest Service Activity Tracking System (FACTS) database, and for the U.S. Bureau of Land Management (BLM) by a separate online record system.

### 1.3. Management Practices and Ecotoxicology

Knowledge about the effectiveness of current forestry practices in protecting downstream resources during chemical applications is limited for Oregon Coast Range watersheds. For example, little information exists to document the effects of no buffer protections under the OFPA for non-fish bearing streams (at the time of sampling), although they comprise up to 70% of the river miles in some watersheds [12,23,24]. Investigations in the neighboring Washington state have led to restrictions in chemical types and buffers on intermittent streams to improve protection of downstream resources [25].

The effect of chemical mixtures used in forestland management, particularly the potential for transport off-site and encounter by non-target species such as invertebrates, fish, and aquatic plants located downstream, is also poorly understood [26,27]. Toxicity benchmarks used to assess risk are derived using LC50 measurements (lethality of compound to 50% of test organisms), yet in the environment, non-target organisms are likely exposed at lower doses and may experience sublethal effects such as disruptions in developmental, hormonal, and reproductive systems [28,29,30,31]. Additionally, research in agricultural systems demonstrates that compound mixtures can exhibit a variety of effects that differ from toxicity of the individual compounds [32,33]. Though the body of research demonstrating effects of chemical mixtures on non-target organisms grows annually, such findings are largely unaccounted for in forestry BMP protocols, creating a knowledge gap in forestry research and management decision making [34]. Moreover, the considerable research focused on behavior of phenoxy herbicides (e.g., 2,4-D) in the forest environment may not adequately describe entry and movement of other commonly used classes of compounds such as triazines (e.g., atrazine) and prevailing mixtures [11].

### 1.4. Monitoring Considerations

Non-point sources of pollution, such as those associated with forest practices, are difficult to trace and hard to quantify due to the transient nature of aquatic contaminants. Cumulative effects and pulsed exposures, however, may be examined by tracking occurrence and bioaccumulation in filter feeding organisms [35,36] and via passive water sampling [37]. Filter feeding bivalves are recognized as sentinel organisms for monitoring water quality, and are frequently used for biomonitoring of chemical exposure because they continually filter water and/or sediment [38]. Limited mobility of sedentary bivalves makes them good indicators of upstream conditions as residues of chemical contamination in their tissues respond to ambient environmental exposure [38,39]. Changes in organismal lipid content throughout the year, which can fluctuate based on reproductive timing [40] and seasonal changes in temperature and food availability [41,42], can influence contaminant uptake and storage [43].

Environmental behaviors and transport pathways of forestland chemicals are determined by a variety of chemical properties including octanol/water partition coefficient (K_ow_), volatility, soil adsorption coefficient (K_oc_), water solubility, and rates of hydrolysis and photolysis. These properties are influenced by environmental mechanisms and ambient conditions including the environmental matrix, temperature, and water chemistry [44]. Many lipophilic compounds (log K_ow_ > 3 and often high K_oc_), which can pass through and accumulate in lipid membranes in aquatic and terrestrial organisms [45], easily sorb to soil and organic matter (high K_oc_) and are more likely to be transported away from primary site of application via particles (i.e., erosion, landslides, or other sediment movement within a watershed) [44]. In contrast, most current-use herbicides are hydrophilic compounds (log K_ow_ < 3; dissolve easily into water), and are typically transported via surface water runoff, groundwater and/or macropore infiltration, and direct application to waterways [34].

### 1.5. Project Goals

Our research sought to elucidate the relationship between current pesticide use in forestland management and its effects on downstream coastal resources. We conducted an empirical study to examine linkages between coastal forest management and forestry-use chemical signatures in estuarine systems by tracking targeted chemical mixtures along a downstream flowpath within Oregon’s coastal watersheds. We measured pesticide tissue concentrations in bivalves to document uptake of a variety of chemicals under a range of active management conditions and prescriptions. We then deployed a series of integrative passive water samplers to monitor organism exposure to hydrophilic compounds that typically go unmeasured in biomonitoring efforts. In addition, we measured in-tissue concentrations of pesticides used outside of IFM to document potential alternate land-uses contributing to bivalve contaminant loads in coastal drainages. Our primary research objectives were to: (1) describe and characterize seasonal differences in bivalve contaminant levels and classes; (2) quantify differences in chemical types, mixtures, and concentrations between bivalve tissue and water samples; and (3) examine variation in chemical exposure based on forestry practices permitted under different management regimes, while noting other sources of detected contamination.

## 2. Materials and Methods

### 2.1. Site Selection

**Figure 1:**
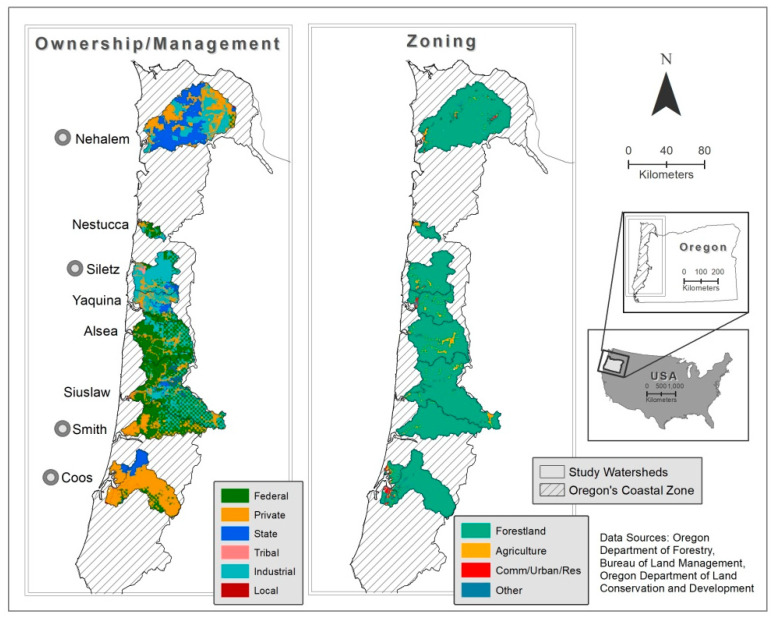
Location of eight watershed areas within the Oregon coastal zone where three species of bivalves were collected for biomonitoring. Colors indicate key land use (ownership and zoning attributes of study watersheds). Circles indicate a subset of watershed areas where passive water sampling was also conducted. Abbreviations: Res/Comm/Indust = zoned for residential, commercial, and industrial uses.

Eight watersheds within Oregon’s coastal zone were selected to encompass a range of forestland management activities across different ownership types (Figure 1). The coastal watersheds were characterized with ArcMap 10.7 to identify key attributes and spatial data regarding federal ownership and land-use zoning under the NWFP, and to characterize state, private, industrial, and tribal ownership areas associated with land-use zoning under the OFPA (Table 1, Figure 1). Sampling sites were selected within watersheds based on the presence, availability, and habitat for target species of bivalves (Table 2), land use (Table 1), and accessibility to stream reaches.

### 2.2. Field Sampling Methods

Given the differences in environmental fate and transport of pesticides both singularly and in mixtures in the forest environment, we designed our sampling methods to explore exposure of filter feeding bivalves to hydrophilic and lipophilic chemicals. We employed biomonitoring and passive water sampling to explore bivalve exposure to both classes of chemicals given their inherent behavioral differences in the environment. 

### 2.3. Biomonitoring of Bivalves

We selected three bivalve mollusk species that inhabit different habitat types within Oregon coastal watersheds: Western pearlshell mussel (*Margaritifera falcata*), softshell clam (*Mya arenaria*), and Pacific oyster (*Crassostrea gigas*). Species attributes such as water salinity tolerances, habitat requirements, feeding type, life history characteristics, life span, and management status differ among these bivalves (Table 2).

Western pearlshell mussels (*M. falcata*), the target species for freshwater habitats, were historically abundant but are increasingly rare with patchy populations due to major population declines throughout their native range [46,47]. Information about the current spatial distribution and abundance of freshwater mussels (including *M. falcata*) in Oregon aquatic systems is limited, and abundance thresholds at sample sites were required to limit potential impacts of this study to the at-risk populations. Several factors were considered in selecting collection sites of *M. falcata*, including: watershed spatial scale (preference toward smaller catchment basins), information about distribution and abundance of current populations, local forestland management practices (sampling areas span a diversity of management types), and access to stream reaches. Three composite samples (five individuals) of *M. falcata* were collected by hand or during snorkel dives [46] from five sites located in four study watersheds during the summer of 2017 (July–August) and three sites in three study watersheds during the spring of 2018 (May–June) (see supplementary material (SM): Figure S1).

Softshell clams (*M. arenaria*), selected as an estuarine species with high tolerance for brackish water, typically inhabit the upper (riverine) region of the estuaries where freshwater drains down from forested watersheds. Exposure of the softshell clams to freshwater was a priority for sample sites, and we collected softshell clams from the uppermost (mesohaline) region of each estuary. Three composite samples (five individuals) of *M. arenaria* were collected from a single site in each of six watersheds during the summer of 2017 (July–August) and eight watersheds during the spring of 2018 (May–June) by digging in the soft mud or sand (Supplementary Materials: Figure S1).

Pacific oysters (*C. gigas*) are non-native bivalves cultured for commercial purposes in the middle (polyhaline) regions of several Oregon estuaries. Composite samples of *C. gigas* (five individuals) were obtained from commercial mariculture operators from two watersheds during summer (2017) and spring (2018) seasons (Supplementary Materials: Figure S1). All wild-stock bivalves (*M. arenaria* and *M. falcata*) were collected under the authority of Oregon Department Fish and Wildlife Scientific Taking Permits (#21207 and #22121).

All sampled bivalves were held in ambient water collected on site (estuarine or freshwater) and transported in a cooler with wet ice to the Applied Coastal Ecology (ACE) Laboratory at Portland State University (Portland, OR; 280 samples) or the Hatfield Marine Science Center (Newport OR; 105 samples) for initial sample processing. Individual bivalves were weighed, shucked, drained, and final shell and tissue wet weights were recorded (Supplementary Materials: Table S5). Samples were composited (five individuals per sample) and frozen at −80 °C, and then homogenized using a CoorsTek mortar and pestle or Waring pulverizor (WSG30 Series), and lyophilized on a HarvestRight or VirTis BenchTop Pro Freeze Drier. Subsamples were sent to the USGS Organic Chemistry Research Laboratory in Sacramento, CA for analysis of pesticides in the bivalve tissues. 

#### Laboratory Analytical Methods

Chromatographic and spectrometric analyses were conducted to determine bivalve tissue concentrations for a wide diversity of fungicides, insecticides, herbicides, and other compounds. Prior to extraction, freeze-dried tissue samples (0.2–0.3 g) were homogenized with sodium sulfate (Na_2_SO_4_) and spiked with ^13^C_12_-*p,p’*-DDE, ^13^C_4_-fipronil, d_4_-imidacloprid, ^13^C_6_-cis permethrin, and d_10_-trifluralin (Cambridge Isotope, Cambridge MA) as recovery surrogates, followed by extraction with 50:50 acetone: dichloromethane (DCM) using a Dionex 200 accelerated solvent extractor (ASE) at 1500 psi and 100 °C. The extract was exchanged into 6 mL of acetonitrile, coextracted matrix interferences were removed with 0.5 g Z-sep+ (Sigma-Aldrich, St. Louis, MO), the eluent was reduced to 0.2 mL, and internal standards were added (d_10_-acenaphthene and d_10_-phenanthrene and d_3_-clothianidin). The bivalve tissue samples were analyzed for a total of 146 pesticides and pesticide degradates (six of which are IFM current-use compounds; see Supplementary Materials: Table S1) using either gas chromatography—tandem mass spectrometry (GC–MS/MS; Agilent 7890 GC coupled to an Agilent 7000 MS/MS operating electron ionization (EI) mode), or liquid chromatography–tandem mass spectrometry (LC–MS/MS; Agilent 1260 bio-inert LC coupled to an Agilent 6430 MS/MS; see [51] for further details). Data for all pesticides were collected in a multiple reaction monitoring (MRM) mode with each compound having one quantifier MRM and at least one qualifier MRM. Ten percent by volume of each raw extract was allowed to evaporate to a constant weight in a fume hood for gravimetric lipid determination to the nearest 0.001 g using a microbalance.

### 2.4. Passive Water Sampling

Integrative passive water sampling was used to characterize pulsed/episodic exposure of the aquatic habitats to contaminants over a longer timeframe [52] because short-term exposure events can easily be missed by grab or composite water sampling efforts. Polar organic chemical integrative samplers (POCISs; developed by the United States Geological Survey (USGS)) capture water soluble organic chemicals from the water column during deployment in a solid phase extraction resin (Oasis HLB sorbent) within two microporous (0.1 micron pore) membranes [52]. Following USGS sampling protocols [52], we deployed the POCIS at sixteen locations during 26–29 March 2019 and retrieved them in identical order 7–10 May 2019 to capture episodic runoff events coinciding with the spring spray events. Exact dates/times and locations of spring spray events were not known, so the timing of deployment and retrieval was determined by the notification of spray events in the FERNS database and documented timing of spray events from previous research in the Coast Range [53]. Documenting spring season exposure was of particular interest because of the reproductive timing of *M. falcata* and *M. arenaria* and their increased vulnerability during early life stages [54,55].

Following retrieval from the field, the POCIS disks were chilled on wet ice, transported to the PSU ACE laboratory, frozen, and shipped to Environmental Sampling Technologies (EST; Missouri) for processing and extraction. Each passive sampler was extracted individually using 25 mL methanol (MSl lot DU 136-US). Following extraction the samplers were blown down over ultra-high pure nitrogen (Air Gas), filtered through glass fiber filter paper (Whatman, GF/D), pooled, blown down again, and quantitatively transferred to 5 mL amber ampules using methanol as the transfer solvent. The ampules were chilled in dry ice and flame sealed. Sample extracts (composites of three POCIS discs) were sent to Anatek Labs (Idaho) for pesticide analysis. Samples were screened for 14 herbicides and one surfactant (all of which are IFM current-use compounds; see Supplementary Materials: Table S2) using either gas chromatography–tandem mass spectrometry (GC–MS/MS) or liquid chromatography–tandem mass spectrometry (LC–MS/MS) (EPA Methods 8151A, 8321A, and 625.1). Resultant concentrations are presented in ng/POCIS, as concentration of chemical per POCIS sample. Detection limits ranged from 3 to 500 ng/POCIS. Maximum and time weighted average concentrations in water could not be calculated due to the dynamics of uptake/degradation of compounds, unknown quantities of total water sampled over the deployment period, and the lack of performance reference compounds. Thus data are used to compare compounds and concentrations across sites (presence/absence and relative concentrations).

### 2.5. Spatial Analysis of Oregon Coast Watersheds

Watershed areas above sampling locations were delineated using StreamStats: Streamflow Statistics and Spatial Analysis Tools for Water-Resources Applications version 4 developed by USGS. Within each watershed boundary we explored planned management activities, physical watershed attributes, and policy/ownership characteristics as factors to explain variation in detections/concentrations among sampling locations. Using StreamStats watershed delineations, physical basin variables were calculated such as average slope, annual rainfall, and area using continuous parameter grids based on 30 m digital elevation models (DEMs). The FERNS database was used to summarize planned management activities within study watersheds. FERNS polygon, line, and point data associated with each activity are accessible through the Oregon Department of Forestry website [56], and the individual detailed notification information is available through a free subscription to the database [57]. Notifications in the FERNS database of aerial herbicide applications active during the sampling period were sorted, imported into ArcMap, and joined with notification polygons. Polygons were clipped to watershed boundaries above sample locations and used to calculate percentage of active notifications within each watershed. Federal forestry activities are available through the FACTS reporting system (USFS land) and the BLM Oregon data library, yet no wide scale reported activities occurred within our study watersheds during the study. Watershed policy/ownership characteristics, summarized as ratios of forestland ownership, were surrogates for land management guiding documents (NWFP, OFPA). Physical watershed characteristics included watershed area, average slope, average annual precipitation, and water temperature at collection (or averaged between deployment and retrieval for passive water samples) derived from StreamStats delineations and field measurements (water temperature).

### 2.6. Statistical Analyses

Organismal lipid content is known to influence bioaccumulation of hydrophobic contaminants in bivalve tissues [58]. Since lipid content can vary annually and among species, we analyzed whether lipid content differed among bivalve species. Differences in lipid content were examined using Kruskal–Wallis nonparametric tests, and pairwise differences were examined using Wilcoxon rank sum tests (R Studio; Version 1.2.5033). Seasonal differences in lipid content within species were explored using Wilcoxon rank sum tests. Lipid-normalized concentrations of chemicals (C_L_) in tissue samples are defined using the following equation:

C_L_= C_i_ ÷ F_L_

where:

C_L_= lipid-normalized concentration;
C_i_ = initial concentration of the chemical in the bivalve tissue (ng/g);
F_L_ = fraction of the tissue that is lipid.

Non-metric multidimensional scaling (NMDS) was used to explore patterns in herbicide detections across POCIS sampling their relationship to watershed variables. This non-parametric approach was used due to its ability to explore patterns independent of underlying distribution assumptions (e.g., non-detect values and skewness). We developed two dimensional ordinations of chemistry profiles detected with sufficient frequency to examine their overlays with land ownership/management and physical watershed variables. Chemistry concertation profiles in NMDS underwent log transformation and Wisconsin double standardization, and the distance matrix was calculated using the Bray–Curtis metric. Correlation matrices were used to visualize relationships between total accumulation in POCIS and watershed variables (see Supplementary Materials: Figure S2). Correlation matrices were used to explore the relationships between both upstream forest management activities and physical watershed characteristics and downstream concentrations of detected herbicides. Least squares linear regressions were used to compare highly correlated variables within categories. Variables were square root transformed to meet regression assumptions.

### 2.7. Quality Assurance/Quality Control

Quality assurance was assessed through the following considerations. During tissue pesticide analysis, the limits of detection (LOD) for tissue contaminants, defined as the value greater than three times the signal-to-noise ratio, were 5–10 ng/g for 0.2 g tissue samples. Additional samples included three laboratory blanks, which did not detect any tissue contaminants, and an acceptable surrogate and matrix spike recovery of 70–130% (all samples were in this range). For the second round of data there were two matrix spikes (acceptable recovery of 70–130%) and two replicates, the relative percent difference between detections was <25%.

Three POCIS discs were composited into one sample per sampling location. At three random sampling locations three replicates (9 POCIS discs) were deployed to assess total method variance. Three field blanks and three laboratory blanks were also used to ensure quality control (QC) throughout deployment, retrieval, and processing. At the three randomly selected replicate sites detections were averaged across the three canisters and the resultant standard deviation was used to assess total method variance.

## 3. Results

### 3.1. Biomonitoring of Bivalves

#### 3.1.1. Bivalve Lipid Content

**Figure 2:**
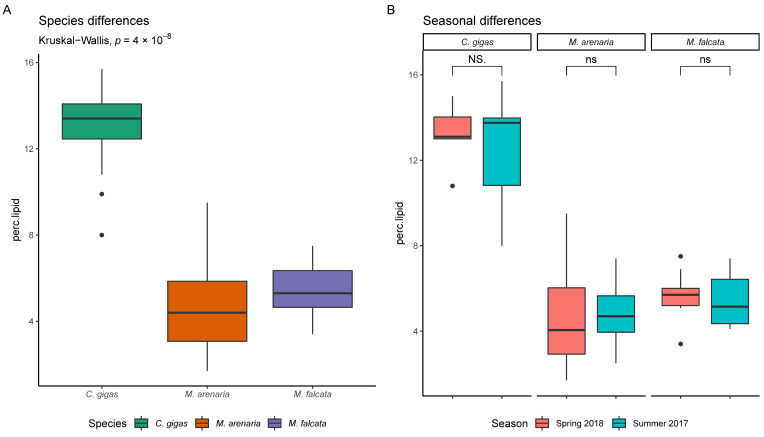
Lipid content of bivalve tissues varied between species (**A**) but not between seasons (**B**) for three species of bivalves that inhabit different areas of the coastal watersheds. ns = not significant.

We collected a total of 385 individual bivalves from 18 watershed sites over two sampling periods (summer 2017 and spring 2018), and the specimens were combined into 77 composite samples of 5 individuals for analysis of pesticide residues (three composite samples per site). Due to low population density at one *M. falcata* collection site (Siletz River), only two replicate composite samples were collected. As expected, shell dimensions and tissue weight varied among species (see Supplementary Materials: Table S5). Bivalve lipid content averaged 6.1% (range 1.7–15.7%) and varied among species (Figure 2A, Kruskal–Wallis, *p* = 4 × 10^−8^) with *C. gigas* having the highest average lipid content, followed by *M. falcata* and *M. arenaria*. In contrast, lipid content did not differ seasonally for any of the species (Figure 2B).

#### 3.1.2. Tissue Pesticide Analysis

**Figure 3:**
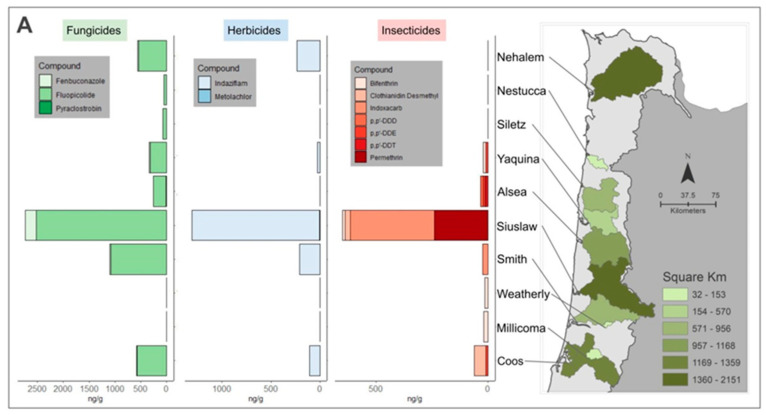

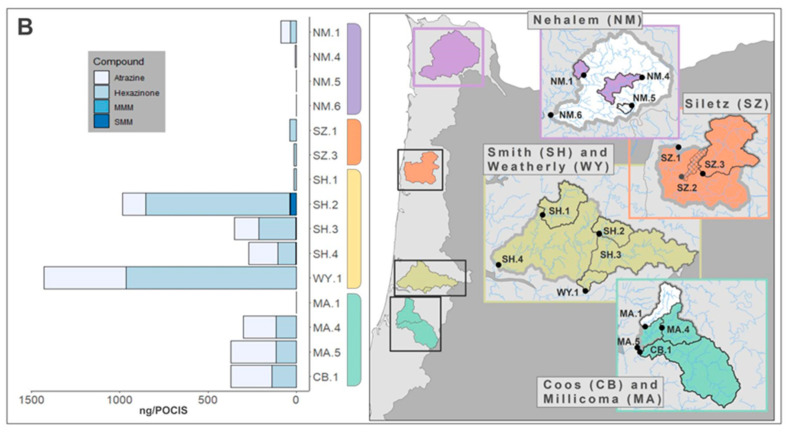
(**A**) Total ng/g (dry weight) detected of insecticides, fungicides, and herbicides in tissues samples across each watershed. Detections varied across sites with Siuslaw watershed displaying consistently elevated levels compared to other watersheds. (**B**) Herbicide detections in polar organic chemical integrative sampler (POCIS) passive water samplers (ng/POCIS). Site abbreviations in the bar chart are associated with mapped sample locations. Detections varied across sites with Weatherly and Smith watersheds displaying elevated levels compared to other watersheds. Hexazinone was the most frequently detected compound, followed by atrazine. Overlaid colors indicate watershed areas and presence of herbicides.

Nine unique pesticides and three pesticide metabolites were detected in bivalve tissues collected during summer 2017 and five were detected in samples collected in spring 2018 across 38% (*n* = 77) of all samples. All study watersheds had at least one detection, though frequency and maximum concentrations varied by season, species, and watershed (Table 3 and Figure 3A). Detections included three fungicides, seven insecticides (including the metabolite), and two herbicides. The fungicide fluopicolide was most frequently detected chemical (23% of samples) and was identified in all three species, followed by the insecticide bifenthrin (8% of samples; Table 3). Bifenthrin, indaziflam (herbicide), metolachlor (herbicide), permethrin (insecticide), and pyraclostrobin (fungicide) were the only detected compounds currently registered for use in Oregon conifer plantations [22], indaziflam (trade name Esplanade F) being the only one used in modern IFM within Oregon [57]. Fluopicolide is registered for use on conifers in neighboring Washington State, but in Oregon is used to control oomycetes in orchards, nursery, or agriculture settings [22]. Legacy insecticides (DDTs), once widely used in Oregon forestlands to control pests [59], were detected in one *M. falcata* and two *C. gigas* samples from summer 2017. Watershed sites exhibited a variety of chemical mixtures (summarized for each watershed in Figure 3A), with the greatest accumulation across all chemical classes in the Siuslaw and Smith, followed by the Coos watersheds. Accumulation of indaziflam, the only pesticide detected in tissue with widespread current use in forestland management, was inconsistent across watersheds, limiting further statistical analyses on watershed and management variables.

**Figure 4:**
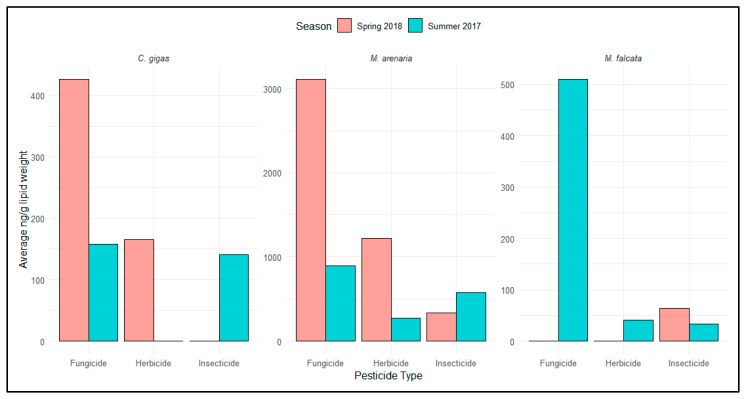
Seasonal changes in mean fungicide, herbicide, and insecticide lipid-normalized concentrations in *C. gigas*, *M. arenaria*, and *M. falcata* tissues. Due to low population sizes, collection sites of *M. falcata* differ by season. *Note the differences in the y-axis scales*.

Lipid-normalization allowed for further distinction of fungicide, herbicide, and insecticide concentrations among bivalve species. The greatest cumulative and average concentrations of all pesticide classes were observed in *M. arenaria*, and the average body burden observed in the species was further exaggerated after accounting for lipid content (Supplementary Materials: Figure S3A,B). Average concentrations of fungicides and herbicides were elevated in spring 2018 for the estuarine bivalves (*M. arenaria* and *C. gigas*). In contrast, average concentrations of fungicides were elevated in the tissues of freshwater bivalves (*M. falcata*) during the summer 2017 (Figure 4), but seasonal comparisons for this species are difficult because collection locations varied between seasons. Average insecticide concentrations were high in the estuarine bivalves during the summer 2017, and the highest insecticide concentrations were observed in freshwater mussels sampled during spring 2018 (Figure 4).

### 3.2. Analysis of Passive Water Samples

#### 3.2.1. POCIS Deployment

Less than two weeks into the POCIS deployment period (2019), southern coast watersheds experienced abnormally severe spring storms from April 6 to 21st that toppled trees and substantially raised river levels, causing widespread flooding and landslides across the region. Damage incurred from flooding, severe weather, and landslides during the significant storm event resulted in a major disaster declaration (FEMA 4452-DR–OR) in 20 July 2019 (https://www.fema.gov/disaster-federal-register-notice/oregon-severe-storms-flooding-landslides-and-mudslides-public). Shortly after the rivers receded, POCIS canisters at two sites (west fork Millicoma River: MA.1; and north fork Smith River: SH.1) were partially stranded on the shore after being deposited there during high waters. The Oasis HLB media in those POCIS canisters was intact so they were processed and reported, yet the duration of time submerged in the river is unknown, so detected chemical concentrations at those locations may under-represent aquatic exposure. Additionally, the membranes in the POCIS canister at the Euchre Creek location (Siletz River: SZ.2) were destroyed at some point during its deployment, with insufficient HLB media remaining for analysis.

#### 3.2.2. POCIS Detections

Four current-use herbicides commonly applied in spring forestland applications (pre-emergent and site preparation treatments) ranged from 1.16 to 936 ng/POCIS and averaged 277 ng/POCIS. Standard deviations at the randomly selected replicate sites were averaged across the three sites for a method standard deviation of 8.06 ng/POCIS (range 0–12.2 ng/POCIS). Detections of the forestry application compounds varied across sampling locations, with the greatest accumulations observed at sites within the Weatherly (predominantly privately managed land) and Smith (mixed federal and private management) watersheds (Figure 3B). Hexazinone was the most commonly detected herbicide (73% of samples) followed by atrazine (60%), sulfometuron-methyl (SMM; 40%), and metsulfuron methyl (MSM; 7%). Field and laboratory blanks returned no detections.

#### 3.2.3. Relationships between Compound Detections and Forestland Management

**Figure 5:**
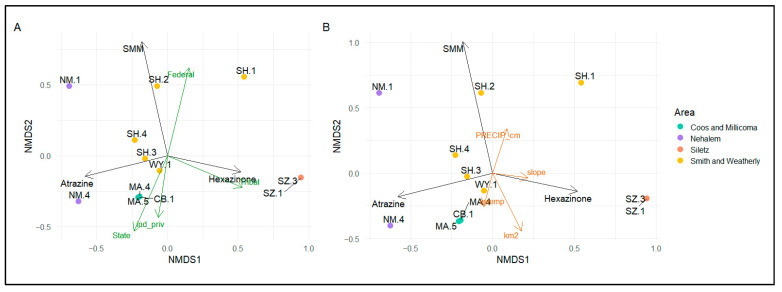
Non-metric multidimensional scaling (NMDS) biplots (stress = 0.017) indicate types of herbicide detections (black vector arrows) across (**A**) site types: ownership/management variables (green vector arrows) and (**B**) associated watershed characteristics: physical watershed variables (orange vector arrows). Similar vector directions of compounds and watershed characteristics indicate associations between the two. Watershed areas are indicated by point color. Detection concentrations were log transformed and the distance matrix was calculated using the Bray–Curtis metric. Abbreviations: ind_priv = industrial and private land ownership, avertemp = average temperature, km^2^ = square kilometers of watershed, PRECIP_cm = annual precipitation in centimeters, SMM = sulfometuron methyl.

NMDS analysis elucidates associations between watershed variables and the compounds detected by POCIS monitoring, with biplots indicating relationships between compounds and forestland ownership based on shared vector direction. Federal ownership appears to be associated with SMM loading, tribal ownership is associated with hexazinone loading, and private and state ownership is associated with atrazine loadings (Figure 5A, stress = 9.62 × 10^−5^). Physical characteristics of the coastal watersheds appeared to have only minor associations with the chemical compound variability (Figure 5B).

**Figure 6:**
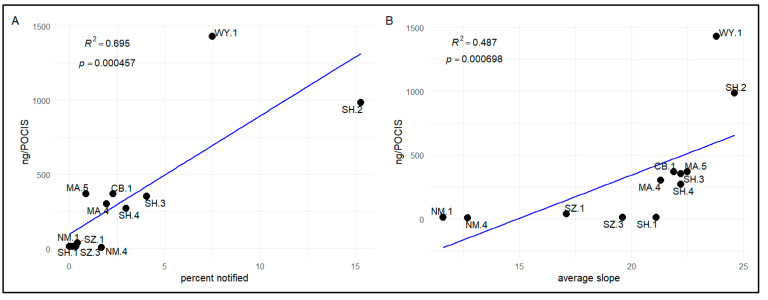
Linear regression indicated that total herbicide load captured in the POCIS was positively correlated with (**A**) active aerial herbicide notifications during deployment window (R^2^ = 0.695, *p* ≤ 0.001) and (**B**) average watershed slope (R^2^ = 0.487, *p* ≤ 0.001). Formulae and results in plots reflect calculations with both variables square root transformed to meet regression assumptions.

Least squares linear regressions of management and physical watershed variables (run separately) revealed that aerial herbicide application (% of watershed) and slope accounted for the greatest variation in total herbicide accumulation in POCIS samplers (Figure 6). Based on simple linear regression, the total herbicide load captured in the POCIS was positively correlated with percentage of active aerial application notifications during the deployment window (R^2^ = 0.694, *p* = 0.0005; Figure 6A), and average watershed slope in upstream catchments (R^2^ = 0.487, *p* = 0.0007; Figure 6B). Negative y-intercept observed in the slope regression relates to high slope catchments (SZ.3 and SH.1) where low concentrations were detected.

### 3.3. Combined Chemical Results and Considerations

This study detected three classes of pesticides (herbicide, insecticide, and fungicide) that exhibit a variety of chemical traits affecting their environmental fate and transport (Table 4). Detected compounds showed wide ranges of water solubility (0.001–33,000 mg/L), octanol/water partition coefficients (Log K_ow_: −1.87–6.91), soil adsorption coefficients (K_oc_; 54–236,610), and leaching potential (−3.89–4.43) (Table 4). Ranges and associated compound detection matrix (tissue or water) were closely aligned with expected behavior in the environment. Passive water samplers detected chemicals that display hydrophilic behavior such as high water solubility, potential for leaching, low bioconcentration factors, and low Log K_ow_ values. With the exceptions of indaziflam and fluopicolide (which straddle the hydrophilic/lipophilic classification, as a consequence of their lower Log K_ow_ values), compounds detected in bivalve tissues are predominantly classified as lipophilic (Table 4). Detected pesticides comprise a variety of registered uses [22] and demonstrate a diversity of modes of action in their respective pesticide classes (Table 4) [60]. Five herbicides (atrazine, hexazinone, indaziflam, metsulfuron-methyl, and sulfometuron-methyl) were the only commonly used forestry-use compounds detected of the eighteen we tested for (Supplementary Materials; Tables S1 and S2). Of the forestry-use compounds we analyzed in both water and tissue samples (atrazine, hexazinone, and sulfometuron-methyl) none were detected in both matrices. Combined results of tissue and water sampling efforts document exposure and uptake of forestry-specific contaminants, and lipophilic compounds from other sources, contributing to pesticide bio-burdens in coastal bivalves.

## 4. Discussion

### 4.1. Interpreting Project Goals and Analyses

This study improves understanding about transport of pesticides applied within Oregon coastal watersheds and subsequent exposure and uptake by bivalves in downstream freshwater and estuarine habitats. In 38% of the bivalve tissue samples, we detected at least one pesticide, with the frequency and maximum concentration of pesticides varying by season, species, and watershed. The greatest tissue accumulation across all chemical classes occurred in the Siuslaw watershed (1780 km^2^) and the Smith watershed (956 km^2^), a coastal sub-basin of the expansive Umpqua drainage system (12,000 km^2^). The Siuslaw and Smith watersheds both encompass a land-use matrix of federal (51.7%; 57.7% of the watershed area respectively) and private (41.9%; 42.6%) forestlands, some agricultural uses (1.4; 2.8%), and small enclaves of rural populations (0.9; 0.1%: Table 1). Our sampling detected a diversity of compounds in downstream waters and bivalve tissues, including three fungicides, seven insecticides, and two herbicides. The fungicide fluopicolide was the compound most frequently detected in bivalve tissues (23.4% of samples), followed by the insecticide bifenthrin (7.8% of samples) and herbicide indaziflam (6.5% of samples). The suite of compounds identified in tissue samples suggests a variety of potential sources may contribute to pesticide burdens, including but not limited to forestland applications, and provide new documentation about types of current-use pesticide contaminants found in Oregon’s coastal bivalves.

Pesticide compounds commonly applied to commercial forestlands were detected by passive water samplers (atrazine, hexazinone, sulfometuron-methyl, and metsulfuron-methyl) and within the tissues of *Margaritifera falcata*, *Mya arenaria*, and *Crassostrea gigas* (indaziflam) in stream and estuarine habitats located considerable distances downstream of the application areas. Water-borne herbicide exposure documented during the spring spray season displayed significant correlations with average watershed slope and planned herbicide activity during the sampling window. These finding suggest a fundamental connection between the spatial patterns of management activities, natural watershed features, and downstream multiscalar ecological processes within the study region.

#### 4.1.1. Seasonal and Species Differences in Contaminant/Exposure Levels

Pesticide contaminants were more frequently detected in bivalve tissues during the summer of 2017 during low runoff conditions, and higher concentrations were detected in the spring of 2018 during high runoff conditions (Table 3). Elevated contaminant levels in spring are expected due to the timing of spring pesticide applications to commercial forestlands and resultant high flow downstream [53,61]. Bivalve tissues frequently exhibit seasonal variability in lipid content due to gametogenesis and reproduction, which can influence the composition and concentration of stored contaminants [62]. However, bivalve lipid content did not vary significantly between summer and spring sampling seasons, but varied significantly among the three bivalve species (Figure 2).

Interspecific comparison of lipophilic compound accumulation among bivalves is challenging due to differences in habitat, salinity, feeding mechanism, reproductive timing, life span, and other life-history characteristics. Lipid normalization allows for comparisons among diverse bivalve species to evaluate differences in tissue pesticide detections between the wet and dry seasons [63,64]. In our samples, lipid normalization inflated existing differences among species’ contaminant burdens, further widening the gap between *M. arenaria* and the other species, while narrowing the range of concentrations between *C. gigas* and *M. falcata* (Supplementary Materials: Figure S3A,B). Elevated pesticide concentrations in *M. arenaria* are likely associated with the location of their preferred habitat at the interface between freshwater and estuarine regions of the watershed (salinity range >5 psu; Table 1) where they are presumably exposed to a diversity of waterborne pollutants carried downstream from multiple points of origin.

#### 4.1.2. Contrast in Compounds Detected in Waters and Bivalve Tissues

Different chemicals detected in tissue versus water samples demonstrate two avenues of chemical fate and transport in the environment, critical in understanding environmental exposure and uptake. The suite of chemical compounds detected in passive water samplers did not overlap with the pesticides detected in tissue samples, with no common compounds detected in both sampling media. These differences are likely attributed to differing biochemical properties and transport pathways (Table 4), suggesting that although forest management activities expose bivalves to herbicide runoff, most current-use herbicides (with the exception of indaziflam) do not accumulate in their tissues. Low bioaccumulation in bivalve tissue is not surprising given the hydrophilic nature of most current-use forestry herbicides. In contrast, the current-use rainfall-activated herbicide indaziflam (Esplanade F [57]), used to control vegetation by ground or aerial application and promoted for its persistence in soil (half-life > 150 days) [21,65], was detected in bivalve tissue in five of eight coastal watershed areas. Widespread detection of indaziflam in bivalve tissue is especially notable as the compound (registered in 2010; Table 4) is classified as both “very toxic to aquatic life” and “very toxic to aquatic life with long lasting effects” by the Globally Harmonized System of Classification Labeling of Chemicals (GHS) [66].

#### 4.1.3. Forestland Management Regimes and Exposure of Bivalves to Pesticides 

We documented accumulation of an array of insecticides, herbicides, and fungicides in bivalve tissue across multiple Oregon Coast Range watersheds. Detections were not consistent across sample locations, hindering statistical analysis relating tissue concentrations with watershed variables (Supplementary Materials; Table S3). Some tissue-detected pesticides are registered for use in plantation forestry management, but others are used in a variety of other crops including orchards, vineyards, and Christmas tree farms [22]. Water protection standards for Christmas tree farms and orchards are not prescriptive, and analysis of upstream rates of usage, prevalence, management activities, and linkages to tissue concentrations remains elusive. According to FERNS notification data, indaziflam is the only detected tissue-bound compound currently applied within the region during vegetation management activities on forestlands [57].

Comparison of POCIS detections among sites indicate that compound accumulation was related to the amount of notified herbicide activity in upstream watersheds (Figure 6A), with types of compounds detected related to ownership/management (Figure 5A). These observations suggest that freshwater and estuarine bivalves in some watersheds may be at risk of pesticide exposure based on upstream forestland management regimes and the pervasiveness of activities. Our NMDS analysis suggests that forestland ownership (a surrogate for pesticide application policy) is related to the types of compounds in water samples (Figure 5A). For example, atrazine (the only herbicide of the four detected in POCIS sampling that is not permitted for use under the NWFP) exhibited a negative association with federal land ownership. In linear modeling, forestland ownership alone was not a strong predictor of chemical exposure, but management practices such as planned forestry herbicide applications influenced aquatic chemical concentrations. In particular, increases in notification of planned aerial herbicide application predicted increases in chemical loads of that pesticide class downstream (Figure 6A).

### 4.2. Additional Factors Affecting Pesticide Exposure and Transport in Coastal Watersheds

#### 4.2.1. Spatial Scale and Complexity of Watershed Drainages

Exploration of downstream pesticide transport following multiple applications allowed us to examine the impact of forestland ownership and management on organismal exposure at the watershed scale. The percentage of coastal watersheds under notice for herbicide spray applications correlated with the concentration of herbicides detected in passive water samples. This relationship indicates a plausible connection between cumulative effects of herbicide applications within a catchment basin and the type and amount of chemical exposure to downstream organisms. However, previous BMP research has highlighted the role of variable abiotic factors, which were not controlled in our study, in understanding offsite movement of chemicals [67,68]. Caldwell and Courter (2020) found that proximity to herbicide application sites followed by rainfall had the greatest influence on herbicide concentration in downstream Oregon coastal waters [67]. Our findings are consistent with these studies and indicate that a rainfall event may result in higher herbicide concentrations in areas with more herbicide applications upstream. Watershed slope was positively correlated with total POCIS accumulation and the best fit for our stepwise linear regression of physical watershed variables (Figure 6B). Watershed slope is consistently an important factor in offsite herbicide transport during site-scale investigations [69] as well as a critical input parameter for modeling pesticide runoff [70,71]. Given that surface runoff is a key process affecting pesticide presence in water [72] the positive association between average watershed slope and the concentrations of herbicides detected by passive water samplers deployed downstream is not surprising.

#### 4.2.2. Ecotoxicity of Pesticide Mixtures and Pulsed Exposures

The wide range of properties associated with detected compounds highlights the variability in chemical partitioning and movement in aquatic ecosystems, and the importance of documenting multiple routes of exposure across scales and timeframes within watersheds. The in-tissue and passive water pesticide mixtures observed in our study align poorly with USEPA toxicity information and established regulatory benchmarks that assume dose–response toxicity of single reference compounds on a small group of selected species [73]. Chemical interactions within complex mixtures (in tank mixes and observed in the field) may result in additive, synergistic, or antagonistic effects on organisms at or below established benchmarks [74,75]. Additional research is needed to better understand organisms’ risks from sublethal exposures based on the documented chemical mixtures of lower doses of forestry (and other) pesticides [11,34]. The discrepancy between pesticide registration requirements and our field observations of chemical mixtures highlights an important knowledge gap and topic for future research.

Organismal age has been identified as an important factor in understanding the impacts of episodic exposure (the commonly observed route of exposure in forestry runoff) to toxicity stressors [76]. Sublethal effects of episodic toxicant exposure can influence population dynamics, especially if exposure occurs to highly sensitive early life stages—juveniles, larva, or during reproduction [68,72,73,77]. Low concentrations of atrazine may alter behavior at non-monotonic dose–responses as observed when short term exposure (72 h) to atrazine (1.5 and 150 μg/L) decreased spatial aggregation (associated with reproduction) by the freshwater mussel *Ellipitio complanata* [78]. Freshwater mussels, which are particularly susceptible to contaminant exposure from surface water during their glochidial stage [79], are among the most sensitive aquatic organisms, and exposure to environmental concentrations of current use pesticides and surfactants have resulted in developmental and genotoxic responses below individual NOEC concentrations of test chemicals [80,81]. Reproductive timing of *M. falcata* is linked to springtime changes in water temperature in Oregon, and glochidia have been observed in the water column from April to mid-June [54]. Our finding of forestry-specific herbicides in the water column during this timeframe suggests that larval mussels in coastal watersheds could be exposed to herbicide mixtures during this sensitive life stage.

#### 4.2.3. Management Practices

Herbicides (such as atrazine) applied to ephemeral stream channels during dry conditions may become mobilized and transported during subsequent rainfall events [11]. Additionally, climatic conditions influence dissipation of atrazine in plantation forestlands, and high rainfall events in temperate locations increase the likelihood of longer persistence in soils and higher offsite mobility [82]. Three detected current-use herbicides (atrazine, indaziflam, and hexazinone) are activated by rainfall for uptake and absorption into the roots of target plants [21]. Reliance on rainfall as the activation mechanism for popular herbicides, combined with a lack of buffer requirements on small type-N and intermittent streams, could explain why increasing compound detections were associated with increased herbicide applications upstream. Atrazine formulation labels typically list buffer restrictions, a 122 m minimum upwind buffer from sensitive vegetation and a 20 m buffer from points where surface water runoff enters perennial or intermittent streams (EPA Reg. No. 35915–4); these are more stringent than OFPA requirements. Indaziflam formulations require a 7.62 m spray buffer around water bodies such as streams or lakes during aerial application (EPA Reg. No. 432–1517). However, no information is available to characterize the level of applicator compliance with these label restrictions.

Vegetated riparian management areas (RMAs) can successfully mitigate contaminant impacts to water quality from runoff and direct infiltration into stream networks, though the minimum size for effective buffers is debated [83,84]. Studies of site-level effects of forestry pesticide application to downstream water quality indicate variability in episodic exposure scenarios, wherein low pulsed concentrations of applied chemicals are observed following application events [67], with most monitoring efforts generally at and below single treatment parcels [23,24,85]. However, earlier research has not specifically investigated movement of chemicals in areas without spray buffers, such as perennial and intermittent stream channels [23,24]. As a result, test conditions and results from previous studies may not fully reflect permitted forestry management practices. Controversy exists between the timber industry and conservation communities around the issue of pesticide use in Oregon’s forestland management, but recent developments indicate a collaborative and cooperative path forward may be on the horizon [19]. A recently adopted Oregon Senate bill (S.B. 1602) provides support and structure for a mediated science-based approach to address shortcomings of OFPA aquatic resource protective measures, but specific approaches to achieve such outcomes have yet to be determined [19].

### 4.3. Caveats and Lessons Learned

Understanding cross-ecosystem linkages, specifically effects of terrestrial activities on riverine and marine species, is a challenging but essential step in designing effective and comprehensive land-sea planning, management, and conservation [3]. Unknown parameters and inherent variability at large spatial scales contribute uncertainty and important limitations or caveats when developing characterizations at the watershed scale [86]. Integrating ecological research such as ours directly into management decisions is complicated by the imperfect picture provided by watershed scale research, in contrast to that provided by controlled laboratory or small-scale field settings with lower inherent variability.

Our efforts to explain the biophysical linkages between coastal watershed forestry practices and bivalve exposure to waterborne toxicants in downstream systems were limited by potentially confounding factors. For example, bivalve sampling across two non-consecutive seasons confounds identification of seasonal differences in pesticide exposure as an underlying factor (Supplementary Materials: Figure S1). Interannual variation in pesticide application levels, timing, and concurrent rainfall are also controlling factors. Similarly, non-forestry sources of contamination can vary annually and spatially. Differences in the habitats, feeding mechanisms, and life-spans of the bivalves studied may contribute to variability in contaminant body burdens. Uncertainty about the specific timing and location of herbicide application activities during the spring spray season required us to extend the deployment of our passive water sampling, making it impossible to calculate realistic time-weighted average water concentrations for the detected herbicides. Consequently, our measurements of forestry herbicides in downstream waters and bivalve tissues are useful to understand compounds’ presence/absence across watersheds and document complex exposure mixtures over time, but do not provide in-water pesticide concentrations to predict toxicity. Differences in the hydrology of the coastal watersheds, and variability in the chemistry of streams and soils, local climates, and the legacy impacts of forestry management practices are only a few of the many uncontrolled factors that may influence our findings. 

## 5. Conclusions

Our study identified that bivalves (and likely other aquatic organisms) in Oregon’s coastal watersheds are exposed to a suite of herbicides commonly used in forestland chemical applications during the spring spray season. Accumulation of measured herbicides in passive water samples was associated with land-use and physical watershed characteristics upstream (frequency of notified herbicide application and average watershed slope). Transient exposures captured in POCIS sampling coupled with varying levels of pesticide residues in bivalves identify specific pesticide compounds, pathways for pesticide transport, and levels of exposure. These findings highlight the need to address management practice effectiveness in controlling transport of potentially harmful compounds throughout the Oregon Coast Range. The precise timing of runoff events remains unknown, and the extent to which such runoff coincides with bivalve reproduction and resultant toxicity exposure in downstream habitats is still speculative. Our study highlights information gaps and research needs to: (1) quantify the extent to which variation in the widths of herbicide spray buffers across stream types function to protect downstream aquatic habitats; (2) explore precise fate and transport of the variety of chemicals used in coastal forest management; and (3) reconcile exposure concentration/duration with chronic or sublethal toxicity endpoints [11]. As scientific understanding of ecotoxicology evolves and new monitoring techniques become available, efforts to understand cross-ecosystem stressors are critical, especially to incorporate ecosystem-based management into watershed-scale or regional land management objectives that go beyond managing for single land uses and individual classes of chemicals.

## Figures and Tables

**Table 1:** Key attributes, zoning, and ownership/management characteristics of the forested watershed basins along the Oregon Coast Range. Abbreviations: Res/Comm/Indust = zoned for residential, commercial, and industrial uses.

Watershed	Watershed Area (sq. Kilometers)	Mean Annual Precip (Centimeters)	Mean Slope (Degrees)	Zoning (%)				Ownership/Management (%)				
				Forestland	Agriculture	Res/Comm/Indust	Other	Federal	State	Industrial/Private	Tribal	Local/Water
Alsea	1168.1	218.7	18.9	93.1	6.3	0.4	0.2	65.2	0.2	34.3	0.1	0.2
Coos	1358.7	178.1	17	92.5	2.5	2.7	2	10.9	13.4	74.9	0	0.8
Nehalem	2150.7	313.2	14.2	96.6	1.5	1.3	0.4	0.8	40.4	58.6	0	0.1
Nestucca	152.8	256.5	13.4	89.9	7.6	2.2	0.4	51.6	3.1	45.3	0	0.0
Siletz	787.4	266.7	17.2	95.3	3.4	0.7	0.5	11.2	3.8	82.2	2.4	0.4
Siuslaw	1779.3	176.3	19.6	96.2	2.8	0.9	0.1	51.7	5.3	42.6	0	0.4
Smith	955.7	185.9	22.2	98.1	1.4	0.1	0.5	57.7	0	41.9	0	0.3
Yaquina	569.8	193.8	17.4	90	6.3	2.2	1.5	15.2	13.2	70.8	0	0.8

**Table 2:** Bivalve species selected for the study exhibit a wide variety of life history characteristics, habitat requirements, salinity tolerances, and life span [46,47,48,49,50]. Abbreviations: psu = practical salinity units, IUCN = International Union for Conservation of Nature.

Species Attributes	*Margaritifera falcata*	*Mya arenaria*	*Crassostrea gigas*
Native Biogeographic Range	Western USA and Canada	East coast of USA, naturalized along west coast	Pacific coast of Asia
Habitat Type	Gravel and cobble substrates	Muddy substrate	Hard or rocky substrate
Water Salinity Preference (psu range)	Freshwater (0)	Upper estuarine; mesohaline, polyhaline (5–30)	Mid estuarine; polyhaline (20–25)
Management and conservation status	Designated as Near Threatened—(IUCN Red List)	Managed as a recreational fishery in Oregon	Commercial mariculture
Life-history Characteristics	Complex life-cycle with demersal glochidia larvae that attach to fish	Complex life-cycle with planktonic veliger larvae	Artificial propagation in hatcheries
Feeding Type	Suspension and deposit feeders	Suspension and deposit feeders	Suspension feeders
Life Span	>100 years	Up to 19 years, generally 10–12 years	Up to 40 years in northern latitudes

**Table 3:** Seasonal variability in the class of pesticides, detected compounds, frequency of detection, and maximum concentrations (ng/g dry weight) observed in *C. gigas, M. arenaria,* and *M. falcata* tissue during summer 2017 and spring 2018. Approximate method detection limits (MDLs) are 5–10 ng/g, ND indicates non-detect. * indicates a metabolite of a parent compound in this class.

Pesticide Class	Detected Compounds	*C. gigas*		*M. arenaria*		*M. falcata*	
		Frequency	Max Conc. (ng/g dry weight)	Frequency	Max Conc. (ng/g dry weight)	Frequency	Max Conc. (ng/g dry weight)
**Summer 2017**							
Fungicides	Fenbuconazole	1/6	16.7	1/18	21.1	0/14	ND
	Fluopicolide	1/6	114.8	4/18	532.5	3/14	191.7
	Pyraclostrobin	0/6	ND	1/18	13.1	0/14	ND
Insecticides	Permethrin	0/6	ND	1/18	238.8	0/14	ND
	Bifenthrin	0/6	ND	2/18	12.7	0/14	ND
	* Clothianidin Desmethyl	1/6	52.2	1/18	24.6	0/14	ND
	*p,p’*-DDT	0/6	ND	0/18	ND	1/14	10.5
	* *p,p’*-DDD	0/6	ND	0/18	ND	1/14	10.9
	* *p,p’*-DDE	2/6	8.7	0/18	ND	1/14	9.8
Herbicides	Metolachlor	0/6	ND	0/18	ND	1/14	7.8
	Indaziflam	0/6	ND	1/18	235.8	1/14	26.6
**Spring 2018**							
Fungicides	Fenbuconazole	1/6	11.8	2/24	215.7	0/9	ND
	Fluopicolide	1/6	264.6	9/24	2421.3	0/9	ND
Insecticides	Bifenthrin	0/6	ND	0/24	ND	4/9	11.6
	Indoxacarb	0/6	ND	2/24	374.6	0/9	ND
Herbicide	Indaziflam	1/6	107.4	2/24	1298.2	0/9	ND

**Table 4:** Detection frequency, current status, and matrix of compounds observed in tissue and water sampling; along with pesticide characteristics that explain environmental behavior [22,60]. * indicates a metabolite of a parent compound in this class.

Compound	Sampling Matrix	Detection Matrix and Frequency	Year Introduced	Active Registration (in OR Forestry)	Pesticide Class	Mode of Action	Solubility—In Water at 20 °C (mg L^−1^)	Log Kow at pH 7, 20 °C	Koc	Groundwater Ubiquity Score (Leaching Potential)	Bioconcentration Factor (Potential Concern)
Atrazine	Tissue, water	Water, 60.0% (*n* = 15)	1957	Yes (yes)	Herbicide	Inhibits photosynthesis (photosystem II)	35	2.7	100	2.57 (Moderate)	4.3 (Low)
Bifenthrin	Tissue	Tissue, 7.8% (*n* = 77)	1984	Yes (yes)	Insecticide	Sodium channel modulator	0.001	6.6	236,610	−2.66 (Low)	1703 (Threshold for concern)
Clothianidin Desmethyl *	Tissue	Tissue, 2.6% (*n* = 77)		Yes (no)	Insecticide *	n/a	n/a	n/a	n/a	n/a	n/a
DDTs	Tissue	Tissue, 3.9% (*n* = 77)	1944	No (no)	Insecticide	Sodium channel modulator	0.006	6.91	151,000	−3.89 (Low)	3173 (Threshold for concern)
Fenbuconazole	Tissue	Tissue, 6.5% (*n* = 77)	1992	Yes (no)	Fungicide	Inhibits sterol biosynthesis in fungi	2.47	3.79		0.63 (Low)	160 (threshold for concern
Fluopicolide	Tissue	Tissue, 23.4% (*n* = 77)	2006	Yes (no)	Fungicide	Delocalizes spectrin-like proteins (novel)	2.8	2.9		3.2	121 (Threshold for concern)
Hexazinone	Tissue, Water	Water, 73.3% (*n* = 15)	1975	Yes (yes)	Herbicide	Inhibits photosynthesis (photosystem II)	33,000	1.17	54	4.43 (High)	7 (Low)
Indaziflam	Tissue	Tissue, 6.5% (*n* = 77)	2010	Yes (yes)	Herbicide	Inhibits cellulose biosynthesis (CB Inhibitor).	2.8	2.8	1000	2.18 (Moderate)	Low risk (based on Kow)
Indoxacarb	Tissue	Tissue, 2.6% (*n* = 77)	1996	Yes (no)	Insecticide	Voltage-dependent sodium channel blocker.	0.2	4.65	4483	0.27 (Low)	77.3 (Low)
Metolachlor	Tissue	Tissue, 1.3% (*n* = 77)	1976	Yes (yes)	Herbicide	Inhibition of VLCFA (inhibition of cell division)	530	3.4	120	2.36 (Moderate)	68.8 (Low)
Metsulfuron- methyl	Water	Water, 6.7% (*n* = 15)	1983	Yes (yes)	Herbicide	Inhibits plant amino acid synthesis	2790	−1.87		3.28 (High)	1 (Low)
Permethrin	Tissue	Tissue, 1.3% (*n* = 77)	1973	Yes (yes)	Insecticide	Sodium channel modulator	0.2	6.1	100,000	−1.62 (Low)	300 (Threshold for concern)
Pyraclostrobin	Tissue	Tissue, 1.3% (*n* = 77)	2000	Yes (yes)	Fungicide	Respiration inhibitor (QoL fungicide)	1.9	3.99	9304	0.05 (Low)	706 (threshold for concern)
Sulfometuron-methyl	Tissue, Water	Water, 40.0% (*n* = 15)	1982	Yes (yes)	Herbicide	Inhibits plant amino acid synthesis	244	−0.51	85	3.92 (High)	(Low)

## Supplementary Materials

The following are available https://www.mdpi.com/2305-6304/9/3/46/s1 as an attachment: Table S1: Bivalve Analyte List, Table S2: POCIS Analyte List, Table S3: Tissue Concentrations, Table S4: POCIS Concentrations, Table S5: Bivalve measurements, Table S6: POCIS site variables, Figure S1: Bivalve sample locations, Figure S2: POCIS correlation matrices, Figure S3: Bivalve lipid normalization pie charts.

## References

[1] Granek E.F., Polasky S., Kappel C.V., Reed D.J., Stoms D.M., Koch E.W., Kennedy C.J., Cramer L.A., Hacker S.D., Barbier E.B. (2010). Ecosystem Services as a Common Language for Coastal Ecosystem-Based Management. Conserv. Biol..

[2] Lester S.E., McLeod K.L., Tallis H., Ruckelshaus M., Halpern B.S., Levin P.S., Chavez F.P., Pomeroy C., McCay B.J., Costello C. (2010). Science in Support of Ecosystem-Based Management for the US West Coast and Beyond. Biol. Conserv..

[3] Álvarez-Romero J.G., Pressey R.L., Ban N.C., Vance-Borland K., Willer C., Klein C.J., Gaines S.D. (2011). Integrated Land-Sea Conservation Planning: The Missing Links. Annu. Rev. Ecol. Evol. Syst..

[4] Stoms D.M., Davis F.W., Andelman S.J., Carr M.H., Gaines S.D., Halpern B.S., Hoenicke R., Leibowitz S.G., Leydecker A., Madin E.M. (2005). Integrated Coastal Reserve Planning: Making the Land–Sea Connection. Front. Ecol. Environ..

[5] Munns W.R. (2006). Assessing Risks to Wildlife Populations from Multiple Stressors: Overview of the Problem and Research Needs. Ecol. Soc..

[6] Department of Land Conservation and Development: Oregon’s Coastal Zone: Oregon Coastal Management Program: State of Oregon. https://www.oregon.gov/lcd/OCMP/Pages/Coastal-Zone.aspx.

[7] Spies T.A., Johnson K.N., Burnett K.M., Ohmann J.L., McComb B.C., Reeves G.H., Bettinger P., Kline J.D., Garber-Yonts B. (2007). Cumulative Ecological and Socioeconomic Effects of Forest Policies in Coastal Oregon. Ecol. Appl..

[8] Johnson S.L., Jones J.A. (2011). Stream Temperature Responses to Forest Harvest and Debris Flows in Western Cascades, Oregon. Can. J. Fish. Aquat. Sci..

[9] Perry T.D., Jones J.A. (2017). Summer Streamflow Deficits from Regenerating Douglas-Fir Forest in the Pacific Northwest, USA. Ecohydrology.

[10] Clark L., Roloff G., Tatum V., Irwin L.L. (2009). Forest Herbicide Effects on Pacific Northwest Ecosystems: A Literature Review. NCASI Tech. Bull..

[11] Norris L.A., Lorz H.W., Gregory S.V. (1991). Forest chemicals. Influences of Forest and Rangeland Management on Salmonid Fishes and Their Habitat.

[12] Spies T.A., Stine P.A., Gravenmier R., Long J.W., Reilly M.J., Mazza R. (2018). Synthesis of Science to Inform Land Management within the Northwest Forest Plan Area: Executive Summary.

[13] Thomas J.W., Franklin J.F., Gordon J., Johnson K.N. (2006). The Northwest Forest Plan: Origins, Components, Implementation Experience, and Suggestions for Change. Conserv. Biol..

[14] Kaplan D.M., White C.G. (2002). Integrating Landscape Ecology into Natural Resource Management.

[15] Forest Ecosystem Management Assessment Team (1993). US Forest Ecosystem Management: An Ecological, Economic, and Social Assessment: Report of the Forest Ecosystem Management Assessment Team.

[16] Boisjolie B.A., Santelmann M.V., Flitcroft R.L., Duncan S.L. (2017). Legal Ecotones: A Comparative Analysis of Riparian Policy Protection in the Oregon Coast Range, USA. J. Environ. Manag..

[17] Adams P.W. (2007). Policy and Management for Headwater Streams in the Pacific Northwest: Synthesis and Reflection. For. Sci..

[18] Oregon Secretary of State Administrative Rules. https://secure.sos.state.or.us/oard/viewSingleRule.action?ruleVrsnRsn=162555.

[19] (2020). Senate Bill 1602, 80th Oregon Legislative Assembly, 2020 Special Session.

[20] US Bureau of Land Management (2010). Vegetation Treatments Using Herbicides on BLM Lands in Oregon (ROD).

[21] Peachy E. (2020). Pacific Northwest Weed Management Handbook [Online].

[22] Pesticide Information Center Online Database (PICOL). https://picol.cahnrs.wsu.edu/.

[23] Dent L., Robben J. (2000). Oregon Department of Forestry: Aerial Pesticide Application Monitoring Final Report.

[24] Louch J., Tatum V., Allen G., Hale V.C., McDonnell J., Danehy R.J., Ice G. (2017). Potential Risks to Freshwater Aquatic Organisms Following a Silvicultural Application of Herbicides in Oregon’s Coast Range. Integr. Environ. Assess. Manag..

[25] Rashin E., Graber C. (1993). Effectiveness of Best Management Practices for Aerial Application of Forest Pesticides.

[26] (2006). Cox Caroline; Surgan Michael Unidentified Inert Ingredients in Pesticides: Implications for Human and Environmental Health. Environ. Health Perspect..

[27] Laetz Cathy A., Baldwin David H., Collier Tracy K., Hebert V., Stark John D., Scholz Nathaniel L. (2009). The Synergistic Toxicity of Pesticide Mixtures: Implications for Risk Assessment and the Conservation of Endangered Pacific Salmon. Environ. Health Perspect..

[28] Greco L., Pellerin J., Capri E., Garnerot F., Louis S., Fournier M., Sacchi A., Fusi M., Lapointe D., Couture P. (2011). Physiological Effects of Temperature and a Herbicide Mixture on the Soft-Shell Clam *Mya Arenaria* (Mollusca, Bivalvia). Environ. Toxicol. Chem..

[29] Renault T., Stoytcheva M. (2011). Effects of Pesticides on Marine Bivalves: What Do We Know and What Do We Need to Know?. Pesticides in the Modern World—Risks and Benefits.

[30] Gunderson M.P., Veldhoen N., Skirrow R.C., Macnab M.K., Ding W., van Aggelen G., Helbing C.C. (2011). Effect of Low Dose Exposure to the Herbicide Atrazine and Its Metabolite on Cytochrome P450 Aromatase and Steroidogenic Factor-1 MRNA Levels in the Brain of Premetamorphic Bullfrog Tadpoles (*Rana Catesbeiana*). Aquat. Toxicol. Amst. Neth..

[31] Tanguy A., Boutet I., Laroche J., Moraga D. (2005). Molecular Identification and Expression Study of Differentially Regulated Genes in the Pacific Oyster *Crassostrea Gigas* in Response to Pesticide Exposure. FEBS J..

[32] Kudsk P., Mathiassen S.K. (2004). Joint Action of Amino Acid Biosynthesis-Inhibiting Herbicides. Weed Res..

[33] Hayes T.B., Case P., Chui S., Chung D., Haeffele C., Haston K., Lee M., Mai V.P., Marjuoa Y., Parker J. (2006). Pesticide Mixtures, Endocrine Disruption, and Amphibian Declines: Are We Underestimating the Impact?. Environ. Health Perspect..

[34] Michael J.L. (2004). Best Management Practices for Silvicultural Chemicals and the Science behind Them. Water Air Soil Pollut. Focus.

[35] Kennish M.J. (1997). Pollution Impacts on Marine Biotic Communities.

[36] Jacomini A.E., Avelar W.E.P., Martinêz A.S., Bonato P.S. (2006). Bioaccumulation of Atrazine in Freshwater Bivalves *Anodontites Trapesialis* (Lamarck, 1819) and *Corbicula Fluminea* (Müller, 1774). Arch. Environ. Contam. Toxicol..

[37] Metcalfe C.D., Helm P., Paterson G., Kaltenecker G., Murray C., Nowierski M., Sultana T. (2019). Pesticides Related to Land Use in Watersheds of the Great Lakes Basin—ScienceDirect. Sci. Total Environ..

[38] National Research Council (1991). Animals as Sentinels of Environmental Health Hazards.

[39] Phillips D.J.H., Rainbow P.S. (1998). Biomonitoring of Trace Aquatic Contaminants.

[40] Siah A., Pellerin J., Benosman A., Gagné J.-P., Amiard J.-C. (2002). Seasonal Gonad Progesterone Pattern in the Soft-Shell Clam *Mya Arenaria*. Comp. Biochem. Physiol. A. Mol. Integr. Physiol..

[41] Haider F., Timm S., Bruhns T., Noor M.N., Sokolova I.M. (2020). Effects of Prolonged Food Limitation on Energy Metabolism and Burrowing Activity of an Infaunal Marine Bivalve, *Mya Arenaria*. Comp. Biochem. Physiol. A. Mol. Integr. Physiol..

[42] Liu W., Li Q., Kong L. (2013). Reproductive Cycle and Seasonal Variations in Lipid Content and Fatty Acid Composition in Gonad of the Cockle *Fulvia Mutica* in Relation to Temperature and Food. J. Ocean Univ. China.

[43] LeBlanc G.A. (1995). Trophic-Level Differences in the Bioconcentration of Chemicals: Implications in Assessing Environmental Biomagnification. Environ. Sci. Technol..

[44] Lee M.J. (2002). Impact of Herbicides on the Forest Ecosystem, Aquatic Ecosystems and Wildlife: The American Experience. Rev. For. Fr. Spec..

[45] Tzilivakis J. Agricultural Substances Databases Background and Support Information. https://sitem.herts.ac.uk/aeru/ppdb/en/docs/5_1.pdf.

[46] Nedeau E., Smith A.K., Stone J., Jepsen S. (2009). Freshwater Mussels of the Pacific Northwest.

[47] Blevins E., Jepsen S., Box J.B., Nez D., Howard J., Maine A., O’Brien C. (2017). Extinction Risk of Western North American Freshwater Mussels: *Anodonta Nuttalliana*, the *Anodonta Oregonensis/Kennerlyi* Clade, *Gonidea Angulata*, and *Margaritifera Falcata*. Freshw. Mollusk Biol. Conserv..

[48] Abraham B.J., Dillon P.L. (1986). Species Profiles. Life Histories and Environmental Requirements of Coastal Fishes and Invertebrates (Mid-Atlantic). Softshell Clam.

[49] Pauley G.B., Van Der Raay D. (1988). Species Profiles: Life Histories and Environmental Requirements of Coastal Fishes and Invertebrates (Pacific Northwest): Pacific Oyster.

[50] Haag W.R. (2012). North American Freshwater Mussels: Natural History, Ecology, and Conservation.

[51] Hladik M.L., Vandever M., Smalling K.L. (2016). Exposure of native bees foraging in an agricultural landscape to current-use pesticides. Sci. Total Environ..

[52] Alvarez D.A. (2010). Guidelines for the Use of the Semipermeable Membrane Device (SPMD) and the Polar Organic Chemical Integrative Sampler (POCIS) in Environmental Monitoring Studies. US Geol. Surv. Tech. Methods.

[53] Oregon Health Authority (2014). Public Health Assessment: Highway 36 Corridor Exposure Investigation.

[54] Allard D.J., Whitesel T.A., Lohr S.C., Koski M.L. (2017). Western Pearlshell Mussel Life History in Merrill Creek, Oregon: Reproductive Timing, Growth, and Movement. Northwest Sci..

[55] Lindsay S., Chasse J., Butler R.A., Morrill W., Van Beneden R.J. (2010). Impacts of Stage-Specific Acute Pesticide Exposure on Predicted Population Structure of the Soft-Shell Clam, *Mya Arenaria*. Aquat. Toxicol..

[56] Oregon Department of Forestry: Maps & Data: About ODF: State of Oregon. https://www.oregon.gov/ODF/AboutODF/Pages/MapsData.aspx.

[57] FERNS—Welcome. https://ferns.odf.oregon.gov/e-notification.

[58] Bruner K.A., Fisher S.W., Landrum P.F. (1994). The Role of the Zebra Mussel, *Dreissena Polymorpha*, in Contaminant Cycling: I. The Effect of Body Size and Lipid Content on the Bioconcentration of PCBs and PAHs. J. Gt. Lakes Res..

[59] Moore D.G., Loper B.R. (1980). DDT Residues in Forest Floors and Soils of Western Oregon, September—November 1966. Pestic. Monit. J..

[60] Lewis K.A., Tzilivakis J., Warner D., Green A. (2016). An International Database for Pesticide Risk Assessments and Management. Hum. Ecol. Risk Assess. Int. J..

[61] Hapke W.B., Morace J.L., Nilsen E.B., Alvarez D.A., Masterson K. (2016). Year-Round Monitoring of Contaminants in Neal and Rogers Creeks, Hood River Basin, Oregon, 2011-12, and Assessment of Risks to Salmonids. PLoS ONE.

[62] Capuzzo J.M., Farrington J.W., Rantamaki P., Clifford C.H., Lancaster B.A., Leavitt D.F., Jia X. (1989). The Relationship between Lipid Composition and Seasonal Differences in the Distribution of PCBs in *Mytilus Edulis* L.. Mar. Environ. Res..

[63] Thompson K.-L., Picard C.R., Chan H.M. (2017). Polycyclic Aromatic Hydrocarbons (PAHs) in Traditionally Harvested Bivalves in Northern British Columbia, Canada. Mar. Pollut. Bull..

[64] Choi J.Y., Yang D.B., Hong G.H., Kim K., Shin K.-H. (2016). Ecological and Human Health Risk from Polychlorinated Biphenyls and Organochlorine Pesticides in Bivalves of Cheonsu Bay, Korea. Environ. Eng. Res..

[65] Kaapro J., Hall J. Indaziflam—A New Herbicide for Pre-Emergent Control of Weeds in Turf, Forestry, Industrial Vegetation and Ornamentals. Proceedings of the 23rd Asian-Pacific Weed Science Society Conference.

[66] National Center for Biotechnology Information Compound Summary for CID 44146693, Indaziflam. http://pubchem.ncbi.nlm.nih.gov/compound/44146693.

[67] Caldwell L.K., Courter L.A. (2020). Abiotic Factors Influence Surface Water Herbicide Concentrations Following Silvicultural Aerial Application in Oregon’s North Coast Range. Integr. Environ. Assess. Manag..

[68] Boyle J.R., Warila J.E., Beschta R.L., Reiter M., Chambers C.C., Gibson W.P., Gregory S.V., Grizzel J., Hagar J.C., Li J.L. (1997). Cumulative Effects of Forestry Practices: An Example Framework for Evaluation from Oregon, U.S.A. Biomass Bioenergy.

[69] Müller K., Trolove M., James T.K., Rahman A. (2004). Herbicide Loss in Runoff: Effects of Herbicide Properties, Slope, and Rainfall Intensity. Soil Res..

[70] Zhang X., Zhang M. (2011). Modeling Effectiveness of Agricultural BMPs to Reduce Sediment Load and Organophosphate Pesticides in Surface Runoff. Sci. Total Environ..

[71] Morselli M., Vitale C.M., Ippolito A., Villa S., Giacchini R., Vighi M., Di Guardo A. (2018). Predicting Pesticide Fate in Small Cultivated Mountain Watersheds Using the DynAPlus Model: Toward Improved Assessment of Peak Exposure. Sci. Total Environ..

[72] Schriever C.A., von der Ohe P.C., Liess M. (2007). Estimating Pesticide Runoff in Small Streams. Chemosphere.

[73] Touart L.W., Maciorowski A.F. (1997). Information Needs for Pesticide Registration in the United States. Ecol. Appl..

[74] Lydy M., Belden J., Wheelock C., Hammock B., Denton D. (2004). Challenges in Regulating Pesticide Mixtures. Ecol. Soc..

[75] Sobiech S.A., Henry M.G. (2002). The Difficulty in Determining the Effects of Pesticides on Aquatic Communities. Biological Response Signatures: Indicator Patterns Using Aquatic Communities.

[76] Gordon A.K., Mantel S.K., Muller N.W.J. (2012). Review of Toxicological Effects Caused by Episodic Stressor Exposure. Environ. Toxicol. Chem..

[77] Perry K., Lynn J. (2009). Detecting Physiological and Pesticide-Induced Apoptosis in Early Developmental Stages of Invasive Bivalves. Hydrobiologia.

[78] Flynn K., Spellman T. (2009). Environmental Levels of Atrazine Decrease Spatial Aggregation in the Freshwater Mussel, *Elliptio Complanata*. Ecotoxicol. Environ. Saf..

[79] Cope W.G., Bringolf R.B., Buchwalter D.B., Newton T.J., Ingersoll C.G., Wang N., Augspurger T., Dwyer F.J., Barnhart M.C., Neves R.J. (2008). Differential Exposure, Duration, and Sensitivity of Unionoidean Bivalve Life Stages to Environmental Contaminants. J. N. Am. Benthol. Soc..

[80] Conners D.E., Black M.C. (2004). Evaluation of Lethality and Genotoxicity in the Freshwater Mussel *Utterbackiaimbecillis* (Bivalvia: Unionidae) Exposed Singly and in Combination to ChemicalsUsed in Lawn Care. Arch. Environ. Contam. Toxicol..

[81] Bringolf R.B., Cope W.G., Mosher S., Barnhart M.C., Shea D. (2007). Acute and Chronic Toxicity of Glyphosate Compounds to Glochidia and Juveniles of *Lampsilis Siliquoidea* (Unionidae). Environ. Toxicol. Chem..

[82] Kookana R., Holz G., Barnes C., Bubb K., Fremlin R., Boardman B. (2010). Impact of Climatic and Soil Conditions on Environmental Fate of Atrazine Used under Plantation Forestry in Australia. J. Environ. Manag..

[83] Mazza R., Olson D. (2015). Heed the Head: Buffer Benefits along Headwater Streams.

[84] Michael J.L., Neary D.G. (1993). Herbicide Dissipation Studies in Southern Forest Ecosystems. Environ. Toxicol. Chem..

[85] Tatum V.L., Jackson C.R., McBroom M.W., Baillie B.R., Schilling E.B., Wigley T.B. (2017). Effectiveness of Forestry Best Management Practices (BMPs) for Reducing the Risk of Forest Herbicide Use to Aquatic Organisms in Streams. For. Ecol. Manag..

[86] Milner-Gulland E.J., Shea K. (2017). Embracing Uncertainty in Applied Ecology. J. Appl. Ecol..

